# Modulatory Effects of a Novel Cyclized Peptide in Reducing the Expression of Markers Linked to Alzheimer's Disease

**DOI:** 10.3389/fnins.2018.00362

**Published:** 2018-06-13

**Authors:** Emanuele Brai, Florian Simon, Antonella Cogoni, Susan A. Greenfield

**Affiliations:** ^1^Culham Science Centre, Neuro-Bio Ltd., Oxfordshire, United Kingdom; ^2^Department of Biotechnology, University of Nîmes, Nîmes, France

**Keywords:** neurodegeneration, Alzheimer's disease, *ex vivo* brain slices, basal forebrain, AChE-derived peptides, α7 nicotinic receptor, amyloid beta, phosphorylated Tau

## Abstract

Despite many studies attempt to identify the primary mechanisms underlying neurodegeneration in Alzheimer's disease (AD), the key events still remain elusive. We have previously shown that a peptide cleaved from the acetylcholinesterase (AChE) C-terminus (T14) can play a pivotal role as a signaling molecule in neurodegeneration, via its interaction with the α7 nicotinic acetylcholine receptor. The main goal of this study is to determine whether a cyclized variant (NBP14) of the toxic AChE-derived peptide can antagonize the effects of its linear counterpart, T14, in modulating well-known markers linked to neurodegeneration. We investigate this hypothesis applying NBP14 on *ex-vivo* rat brain slices containing the basal forebrain. Western blot analysis revealed an inhibitory action of NBP14 on naturally occurring T14 peptide, as well as on endogenous amyloid beta, whereas the expression of the nicotinic receptor and phosphorylated Tau was relatively unaffected. These results further confirm the neurotoxic properties of the AChE-peptide and show for the first time in an *ex-vivo* preparation the possible neuroprotective activity of NBP14, over a protracted period of hours, indicating that T14 pathway may offer a new prospect for therapeutic intervention in AD pathobiology.

## Introduction

Alzheimer's disease (AD) is a chronic neurodegenerative disorder and is regarded as the most common form of dementia. AD is a multifactorial pathology which progressively compromises integrity and functionality of several brain areas and leads, in its late stage, to cognitive decline (Albert et al., 2011; Jack et al., 2011; McKhann et al., 2011; Dubois et al., 2014). The major neuropathological features characterizing the disease include extracellular senile plaques (SPs) and intracellular neurofibrillary tangles (NFTs) (Braak and Braak, 1991; Inestrosa et al., 2004, 2005; Braak and Del Tredici, 2011; Brai et al., 2016; Arendt et al., 2017; Brandt and Bakota, 2017). The SPs, distributed in the brain parenchyma, are constituted by the deposition of several misfolded proteins, including amyloid beta (Aβ), acetylcholinesterase (AChE), and α7 nicotinic acetylcholine receptor (α7-nAChR) (Talesa, 2001; Murphy and LeVine, 2010; Lombardo and Maskos, 2015). The main component of the NFTs is the microtubule associated protein Tau, which in its hyper phosphorylated state tends to aggregate and form fibrillary structures (Stoothoff and Johnson, 2005).

However, despite the clear involvement of SPs and NFTs to AD progression, their presence does not elucidate the pivotal mechanisms underlying neuronal death: hence increasingly studies are challenging the most widespread theories on the primary causes characterizing AD, in particular the “amyloid theory” (Morris et al., 2014; Herrup, 2015; De Strooper and Karran, 2016; Scheltens et al., 2016). An alternative hypothesis (Greenfield, 2013) proposes that the basic events leading to neurodegeneration occur in an interconnecting hub of nuclei formed by distinct neuronal populations, identified as “global neurons” (Woolf, 1996). These cells are distributed from the basal forebrain to brainstem (Arendt et al., 1992; Auld et al., 2002; Mesulam, 2004; Mesulam et al., 2004; Schliebs and Arendt, 2011; Schmitz et al., 2016) and project to diverse higher brain areas, such as olfactory system, cortical mantle and hippocampal region (Mesulam et al., 1983a,b; Ballinger et al., 2016).

Although these nuclei are heterogeneous with respect to transmitters, morphology, and distribution, they all express acetylcholinesterase, which has been suggested for many decades to have a non-hydrolytic activity (Appleyard, 1992; Soreq and Seidman, 2001; Silman and Sussman, 2005; Greenfield, 2013; Garcia-Ratés et al., 2016). For instance, independent of its enzymatic action, AChE can modulate calcium (Ca^2+^) influx (Soreq and Seidman, 2001; Greenfield, 2013; Zimmermann, 2013; Garcia-Ratés et al., 2016), which subsequently can trigger trophic or toxic mechanisms depending on its dose (Bon and Greenfield, 2003; Greenfield et al., 2004), exposure time (Day and Greenfield, 2003) and, of most relevance to AD, the age of the brain in question (Eimerl and Schramm, 1994; Riascos et al., 2011). Specifically, the non-classical action of this enzyme could be excitotoxic due to a 30mer fragment (T30), cleaved from its C-terminus, which is respectively composed of a bioactive sequence, T14, (Greenfield and Vaux, 2002) and one inactive portion, T15 (Bond et al., 2009). The AChE-derived peptide can enhance cytoplasmic Ca^2+^ concentration and trigger downstream molecular cascades by initially binding to an allosteric site of the alpha7 nicotinic acetylcholine receptor (Greenfield and Vaux, 2002; Greenfield et al., 2004; Bond et al., 2009), thereby acting as a positive allosteric modulator (Garcia-Ratés et al., 2016). We have previously demonstrated that the AChE-peptide is increased in AD brains (Garcia-Ratés et al., 2016) and its interaction with the α7-nAChR can elicit neurodegenerative-like events, such as reduction of neuronal activity monitored in real-time (Badin et al., 2016) and alteration of protein levels, including the nicotinic receptor itself, Aβ, APP, p-Tau, and GSK3 (Garcia-Ratés et al., 2016; Brai et al., 2017), thus compromising cell viability (Garcia-Ratés et al., 2016). A distinguishing feature of the “global neurons” is that they have a different embryological origin and, unlike all other cells, have retained robust sensitivity to trophic agents (Woolf, 1996): hence in the event of neuronal insult these specific cells will mobilize developmental mechanisms, where calcium signaling plays a crucial role and can display excitotoxic action in mature neurons (Eimerl and Schramm, 1994). The toxic influx of calcium can subsequently be continued by a misplaced “compensatory” AChE release from extant cells (Greenfield et al., 2008; Garcia-Ratés et al., 2013, 2016) which can further lead to a subsequent T14 production that, upon its binding to the nicotinic receptor, might induce a persistent Ca^2+^ permeability (Greenfield et al., 2008; Garcia-Ratés et al., 2013, 2016), promoting a positive feed-forward cascade and contributing to excitotoxicity and progressive cell loss.

In addition, the AChE-peptide shares a sequence homology with Aβ (Cottingham et al., 2002; Greenfield, 2013; Garcia-Ratés et al., 2016), which also binds, as the AChE-peptide, to the α7-nAChR (Wang et al., 2000a,b, 2003). It is possible that these molecules may participate to common pathways by interacting synergistically at the same receptor target.

Hence, we have also aimed to demonstrate that T14 and the alpha-7 nicotinic receptor form a protein-protein complex.

Moreover, the detrimental processes mediated by T30 are all reversed by a novel α7-nAChR allosteric modulator, NBP14, namely a cyclized form of the active sequence, T14 (Badin et al., 2016; Garcia-Ratés et al., 2016). In this study, we further explore the actions of the AChE-derived peptides using a novel approach based on *ex-vivo* rat brain slices and previously described (Brai et al., 2017, 2018). Specifically, we aim to investigate the potential effects of NBP14, compared to T30, in modulating the expression pattern of the endogenous T14, its receptor target and the familiar AD markers, Aβ, and p-Tau.

## Materials and methods

### Peptides

The peptides used in this study (Table 1) were synthesized by Genosphere Biotechnologies (Paris, France) as previously described (Garcia-Ratés et al., 2016) and covered by patent number GB1505239.2 and WO 2015/004430. All compounds were applied at a concentration of 2 μM.

**Table 1:** Amino acid sequence of the compounds used in this work.

**Molecule**	**Sequence**
T30	KAEFHRWSSYMVHWKNQFDHYSKQDRCSDL
NBP14 (cyclic T14)	AEFHRWSSYMVHWK
T15	NQFDHYSKQDRCSDL

### Animals

In this work, postnatal day 14 (P14) wild type male Wistar rats were used to test four different treatments, presenting two conditions each (Table 2). P14 rats were chosen to expand previous results describing changes in protein expression and neuronal network activity observed in age-matched animals (Badin et al., 2016; Brai et al., 2017). The number of animals used in each treatment is indicated in the figure legends. In addition, 4 wild type male Wistar rats (1 P7, 2 P14, and 1 P21) were used to perform co-immunoprecipitation assay. The procedure on animal experimentation was approved and performed in accordance with the UK Home Office regulations (Animals (Scientific Procedures) Act, 1986), following the “Schedule 1” indications, and European guidelines 2010/63/EU.

**Table 2:** Treatments and related conditions used in this study.

**Treatments**		
**Condition 1**		**Condition 2**
Ctrl	vs.	T30
Ctrl	vs.	NBP14
T30	vs.	NBP14+T30
Ctrl	vs.	T15

### Experimental procedure: brain dissection, slicing, and incubation

**Figure 1:**
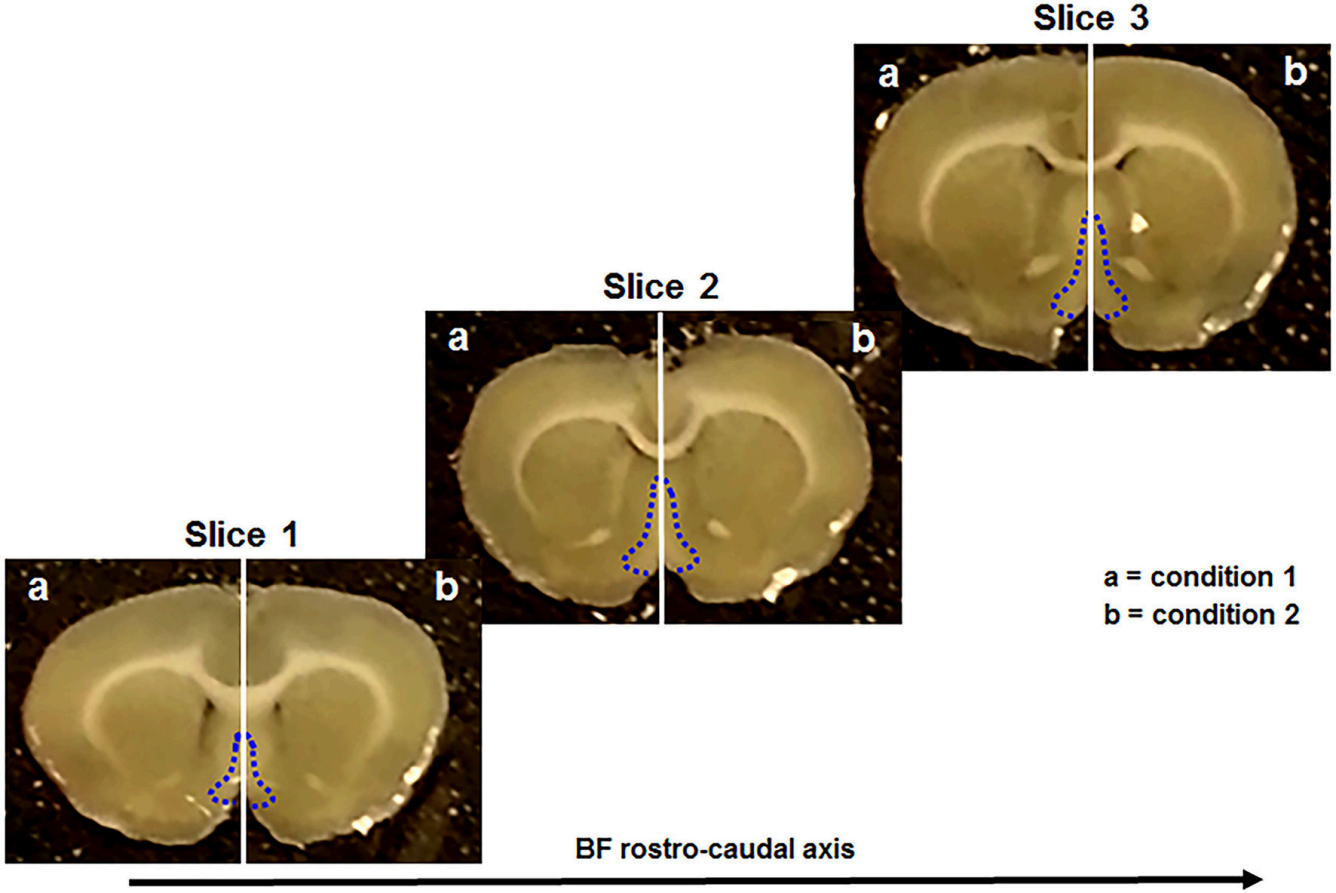
Coronal brain sections used in this study. They include the rostral (slice 1), the intermediate (slice 2), and the caudal (slice 3) portion of the basal forebrain (BF) (blue dotted line). Each slice is divided in two complementary halves (white vertical line) and treated with different conditions (a, b).

All these steps were performed as previously described (Brai et al., 2017, 2018). Briefly, anesthesia was induced upon isoflurane administration (100% w/w) and its proper level was established by the absence of the pedal withdrawal reflex. After, the animals were decapitated and the brain was rapidly removed and kept in ice-cold “slicing” artificial cerebrospinal fluid (aCSF). Subsequently, the brain was vibratome-sliced and three consecutive sections (300 μm thick) were collected within the following stereotaxic coordinates: Bregma 1.20–0.20 mm (Paxinos, 1998). These sections contain the rostral (Figure 1, slice 1), intermediate (Figure 1, slice 2), and caudal (Figure 1, slice 3) portion of the basal forebrain (Figure 1, blue dotted areas). In particular, the major BF structures comprised along the rostro-caudal axis were the medial septum (MS), the diagonal band of Broca (DBB), and the substantia innominata (SI), including the nucleus basalis of Meynert (NBM). Afterwards, each slice was divided along the midline (Figure 1, vertical white lines) providing two complementary halves of the same anatomical plane. Successively, three serial hemisections (Figure 1, slice 1-3 a) were incubated, for 5 hours (h), with condition 1 and their corresponding counterparts (Figure 1, slice 1-3 b) with condition 2, as previously described (Brai et al., 2017, 2018). Depending from the treatment, the brain tissue was incubated with “recording” aCSF alone or enriched with the aforementioned peptides (Table 1), in order to test different conditions (Table 2). The working concentrations (mmol) of the two aCSFs, previously described (Badin et al., 2013; Brai et al., 2017), are the following: “slicing” aCSF: 120 NaCl, 5 KCl, 20 NaHCO_3_, 2.4 CaCl_2_, 2 MgSO_4_, 1.2 KH_2_PO_4_ and 10 glucose; 6.7 HEPES salt and 3.3 HEPES acid; pH: 7.1. “Recording” aCSF: 124 NaCl, 3.7 KCl, 26 NaHCO_3_, 2 CaCl_2_, 1.3 MgSO_4_, 1.3 KH_2_PO_4_, and 10 glucose; pH: 7.1. After the incubation, the hemisections were homogenized with lysis buffer containing protease (Roche complete PIC, 04693116001, USA) and phosphatase (Fisher, cat # 1284-1650, USA) inhibitors diluted in PBS 1x. Next, the tissue lysate was centrifuged at 1,000 g for 5 minutes (min) at 4°C and the supernatant transferred into a new tube and stored at −80°C until use.

The advantage of this methodology is that it offers (1) the opportunity of investigating medium-term responses (hours) upon stimulation with different compounds; (2) the possibility to simultaneously compare the responses evoked by different conditions all within the same anatomical plane and animal. A key issue with this methodology is the maintenance of the tissue viability. Using an identical preparation (slicing procedure, aCSFs composition, recovery time, and peptides used) we have previously demonstrated with optical imaging and electrophysiology techniques the viability of *ex-vivo* brain sections including the basal forebrain (Badin et al., 2013, 2016). In particular, optical imaging showed that the evoked cellular response upon stimulation is detectable with no decrement throughout the experiment. This evidence is further supported by long field potential electrophysiology recordings indicating that signal magnitude of the investigated area is not changed during the whole experiment, thereby demonstrating the integrity of the cell membrane and consequently the functionality of the tissue. These observations are in line with other studies assessing the slice viability through long field potential recording in hippocampal sections (Yu et al., 2013).

### Co-immunoprecipitation assay

Immunoprecipitations were performed from both *ex-vivo* brain slices and whole brain lysates. The latter were obtained after homogenizing the tissue with lysis buffer (Thermo Scientific, cat # 88804) enriched with phosphatase (Fisher, cat # 1284-1650, USA) and protease inhibitors (Roche complete PIC, cat # 04693116001, USA). Subsequently, the samples were sonicated for 10–15 s (Homogenizer Status x 120, cat # 60404, Germany) and centrifuged at 16,300 g for 30 at min 4°C. The resulting supernatant was transferred into a new tube and used to determine protein concentration and subsequently proceed with immunoprecipitation (IP) with 1–1.2 mg of protein content. For the IP on brain slices was used a protein concentration of 500 μg. IP procedure was done following the protocol and using the reagents of a purchased kit (Thermo Scientific, cat # 88804, USA). Briefly, a pre-clearing step was performed adding the lysate to 10 μl of pre-washed magnetic beads and placed at 4°C for 1 h on the rotating wheel. Next, the tubes were quickly spun and the beads separated from the samples using a magnetic rack. The supernatant was transferred in a new tube where the following primary antibodies were added to form the immune complex: goat anti-α7-nAChR (10 μg/1.2 mg of lysate; Santa Cruz Biotechnology, # sc58607, USA), rabbit anti-T14 (10 μg/1.2 mg of lysate; Genosphere, France). As negative control plain beads were added to the homogenate. After, the lysates were incubated for 2 h at 4°C on the rotating wheel. Next, the tubes were quickly spun and the lysates were added into new tubes containing 30 μl of pre-washed magnetic beads and incubated at the same conditions. After, the tubes were quickly spun and placed in a magnetic rack to collect the beads (bound to the immune complex) and remove the unbound sample. Then, the beads were rinsed twice with wash buffer and once with ultra-pure water (Thermo Scientific, cat # 10977-035, USA). Next, 60 μl of elution buffer, containing 4x Laemmli Sample Buffer (Biorad, cat #161-0747 USA), diluted four-fold with ultra-pure water and 2-mercaptoethanol (Biorad, cat # 161-0710, USA), were added to the beads and mixed at room temperature (RT) for 10 min. Then the beads were magnetically separated and the supernatant containing the target protein collected for WB analysis.

### Western blot

Protein concentration assay, sample preparation and western blot analysis were performed as recently described (Brai et al., 2017). Briefly, the protein content in each sample was measured through a standardized protocol, following the manufacturer's indications (Pierce 660 nm protein assay, Thermo Scientific, cat # 22660). Subsequently, regarding the brain slices, the aliquots for WB analysis were prepared mixing equal amount of proteins (10μg/μl), with 4x Laemmli Sample Buffer (Biorad, cat #,161-0747 USA) and 2-mercaptoethanol and finally heated at 95°C for 5 min. After, the samples were loaded on 4–15% precast gels (Bio Rad, cat # 456-1084, USA) for the electrophoresis step and successively transferred on PVDF membranes (Immobilon-P, Sigma-Aldrich, cat # P2938, USA). Next, the membranes were temporarily stained with Blot FastStain (G-Biosciences, cat # 786-34, USA), following the manufacturer's instructions, in order to determine the transfer quality and the total protein content, which was imaged with a CCD camera (G-Box, Syngene, Cambridge, UK) and used as loading control for the statistical analysis, as previously described (Aldridge et al., 2008; Zeng et al., 2013; Collins et al., 2015). Then, the membranes were destained with warm distilled water and incubated with a 5% milk solution, prepared mixing TBS Tween 0.05% (TBST) (Sigma-Aldrich, cat # P9416, Germany) with blotting grade blocker (Bio Rad, cat # 170-6404, USA), for 1 h at RT, with gentle agitation. Afterwards, the blots were rinsed three times for 5 min with TBST and incubated overnight at 4°C with gentle agitation with primary antibodies, all diluted 1:1,000 except Gapdh 1:10,000. The antibodies were rabbit anti-T14 (Genosphere, France), rabbit anti α7-nAChR (Abcam, ab10096, UK), rabbit anti-amyloid beta (Cell Signaling, cat # 8243, USA), mouse anti-phosphorylated Tau (Thermo Fisher, MN1020, UK), and rabbit anti-Gapdh (Abcam, ab181602, UK). T14 and p-Tau antibodies were diluted in 1% blocking solution (mixing TBST and blotting grade blocker), whereas Aβ and α7 receptor following the manufacturer's datasheet. The next day, the membranes were rinsed 5 times for 5 min with TBST and incubated at RT for one h with gentle agitation with secondary antibodies both HRP conjugated, goat anti-mouse (1:2,000) (Sigma-Aldrich, A9309, Germany) and goat anti-rabbit (1:5,000) (Abcam, ab6721, UK). Both antibodies were diluted in TBST. After the incubation, the blots were washed six times for 5 min with TBST and finally for 10 min with TBS 1x before detecting the protein bands using the chemiluminescence revelation system ECL (Bio Rad, cat # 170-5061, USA) through the manufacturer's instructions and imaged with the CCD Camera apparatus.

### Image processing and statistical analysis

Membrane images representing the total protein content (Figures 3A,C,E,G, 4A,C,E, 5A,C,E, 6A,C,E) were used as loading control (LC) as previously described (Aldridge et al., 2008; Zeng et al., 2013; Collins et al., 2015). The band intensity was determined with ImageJ software (NIH, USA) as described in the following link: https://www.youtube.com/watch?v=JlR5v-DsTds. Briefly, a rectangular box was drawn around each lane of the total protein blot and then the relative optical density (number of pixels) within that area was calculated. The values obtained from all the lanes were then averaged and used to calculate the coefficient of variation (CV). Low CV values indicate consistency in the sample loading. After the incubation with primary antibodies, the expression of the protein of interest (POI) was quantified using the same approach, i.e., drawing a rectangle around the band and evaluating the optical density. Next, the intensity values of the LC and POI were processed, using Excel 2013 (Microsoft, USA), dividing the POI by the LC in order to standardize the samples inter-variability. The resulting values provided a readout of the POI expression after testing two conditions (e.g., Ctrl vs. NBP14). Subsequently, the control group (condition 1) and NBP14 group (condition 2) values were normalized, dividing each of them by the mean of the control group. For this reason the “condition 1” in each graph has a baseline value of 1 while the “condition 2” values show the relative change against the reference group. Image processing (adjustment of brightness and contrast) was uniformly applied to the blots using Photoshop Cs6 software (Adobe Systems Inc., San Jose, CA, USA) in order to decrease the background noise. After normalization, the data were statistically analyzed and plotted with GraphPad Prism 6 (GraphPad Software Inc., San Diego, CA, USA).

Variations in protein expression associated to the different conditions and anatomical planes (two independent variables) were evaluated using two-way ANOVA. In addition, when ANOVA revealed a main effect between variables, a two-tailed paired Student's *t*-test was used as post-hoc analysis to assess site-specific protein changes between conditions within the same anatomical level. The cropped blots showed in the figures are chosen from one animal per treatment and might not immediately reflect the data plotted in the graphs. In Supplementary Figures 1–5 are provided the uncropped blots indicating the proteins of interest represented in the main figures. ANOVA and Student *t*-test values are indicated both in the results and figure legends, but the asterisks in the graphs refer only to the Student *t*-test analysis. Data were considered significant when *p*-value < 0.05 and are indicated as mean ± SEM. Statistical significance: ^*^p < 0.05; ^***^p < 0.001.

## Results

### T14 and α7-nAChR molecular interaction

**Figure 2:**
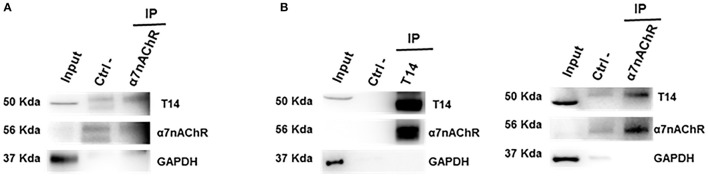
T14 and α7-nAChR directly interact. **(A)** Western blot analysis on immunoprecipitated lysate from *ex-vivo* brain sections (from seven animals) shows a protein-protein complex between the AChE bioactive peptide and the nicotinic receptor (*n* = 2 independent pulldowns). **(B)** Immunolabeling on IP samples from whole brain homogenates confirms the interaction between the peptide and the receptor (*n* = 4 independent experiments). In **(A**,**B)** Gapdh is used as loading control for the input and to identify any contamination in the immunoprecipitated samples.

Co-IP assay was performed to address whether the endogenous AChE-peptide and its target receptor form a molecular complex. To confirm this hypothesis the lysates from control *ex-vivo* brain slices (Figure 2A) and whole brain tissues (Figure 2B) were used to perform co-immunoprecipitation experiments. We observed that T14 and α7-nAChR show a reciprocal interaction (Figure 2A and Supplementary Figure 1). The protein-protein interaction was confirmed by Co-IP on whole brain lysate (Figure 2B).

### Effects of AChE-derived peptides on endogenous T14

**Figure 3:**
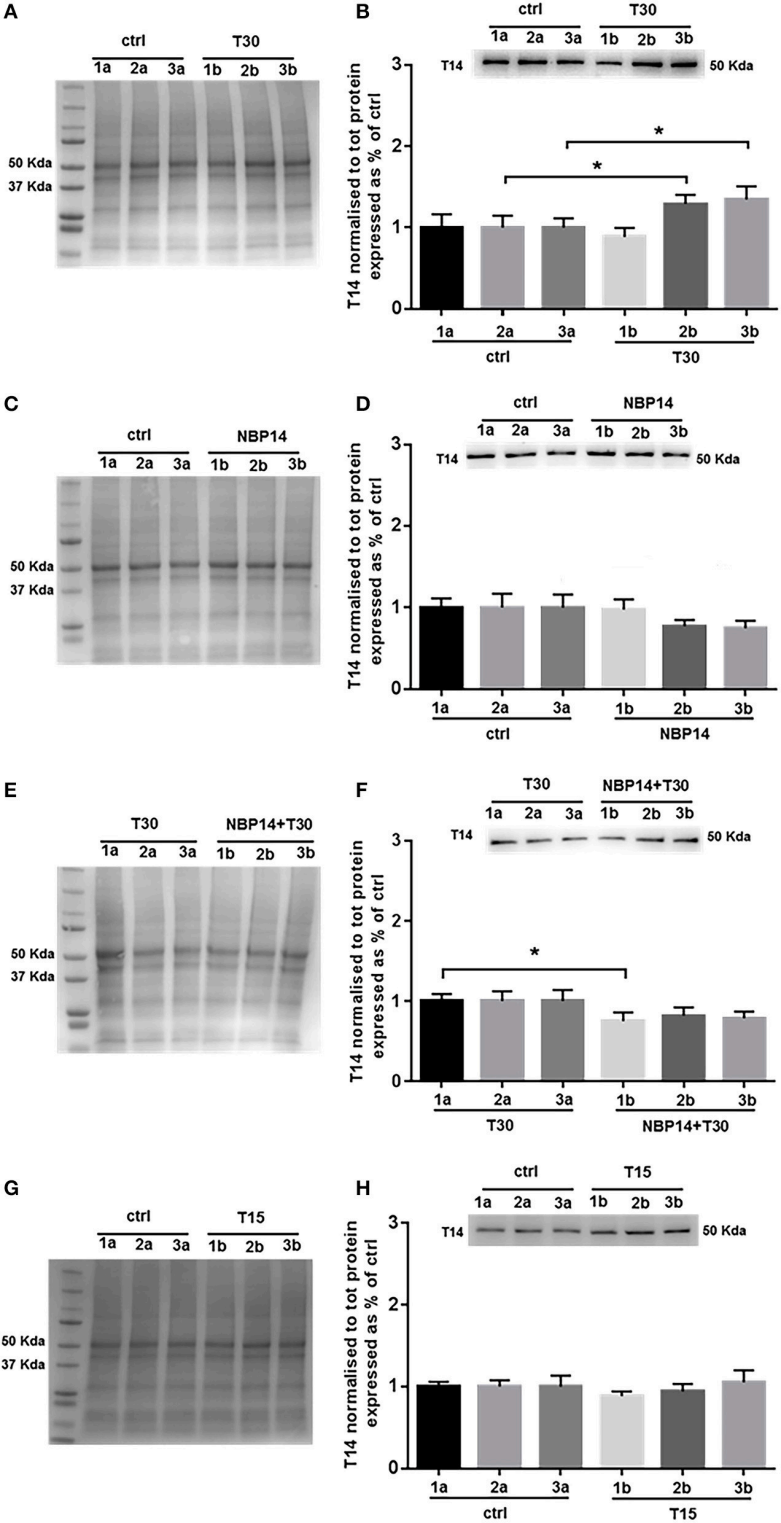
Endogenous T14 is differently modulated by distinct treatments. **(A,C,E,G)** Representative total protein staining used as loading control. **(B,D,F,H)** Representative blots and bar graphs indicating T14 levels along the three BF subdivisions. **(A)** Total protein membrane subsequently immunoblotted for T14. **(B)** T30 exposure strongly affected the endogenous peptide expression across conditions and anatomical planes [*F*_(5, 55)_ = 4.669, *p* = 0.0013, two-way ANOVA]. A significant T14 enhancement was observed in the intermediate and posterior subdivisions (2b and 3b) over their controls (2a and 3a) (slice 2, *p* = 0.0171, *t* = 2.806; slice 3, *p* = 0.0273, *t* = 2.544, two tailed paired *t*-test), whereas the anterior region showed similar levels between the two portions (1a and 1b) (slice 1, *p* = 0.4736, *t* = 0.7421, two tailed paired *t*-test). **(C)** Total protein membrane subsequently immunoblotted for T14. **(D)** Despite the decreasing trend, NBP14 did not significantly alter T14 expression across BF areas and conditions [*F*_(5, 70)_ = 2.074, *p* = 0.0790, two-way ANOVA]. **(E)** Total protein membrane subsequently immunoblotted for T14. **(F)** The interaction between conditions and anatomical planes strongly modulated T14 [*F*_(5,35)_ = 3.370, *p* = 0.0137, two-way ANOVA]. A substantial decrease was detected in the rostral double treated hemisection (1b) over its matching contralateral side (1a), while in the other two sections no major changes were observed (slice 2 and 3) (slice 1, *p* = 0.0109, *t* = 3.435; slice 2, *p* = 0.0779, *t* = 2.064; slice 3, *p* = 0.1538, *t* = 1.599, two tailed paired *t*-test). **(G)** Total protein membrane subsequently immunoblotted for T14. **(H)** T15 did not induce any change in T14 levels [F_(5, 35)_ = 0.6190, *p* = 0.6861, two-way ANOVA]. Protein changes were indicated as mean ± SEM. *n* = (hemisections per condition, rats) in **(A,B)** are (36, 12); in **(C,D)** (45, 15); in **(E,F)** (24, 8); in **(G,H)** (24, 8). ^*^p < 0.05.

Western blot analysis was performed to determine whether T14 levels could change in relation to different treatments and in a site-dependent manner along the BF rostro-caudal axis. Upon T30 administration a significant interaction between treatment and region was observed [Figure 3B; *F*_(5, 55)_ = 4.669, *p* = 0.0013, two-way ANOVA]. Moreover, *post-hoc* analysis revealed a similar T14 expression in the anterior portion between conditions, while a significant increase was detected in the intermediate and posterior regions over their control counterparts (Figure 3B; slice 1, *p* = 0.4736, *t* = 0.7421; slice 2, *p* = 0.0171, *t* = 2.806; slice 3, *p* = 0.0273, *t* = 2.544, two tailed paired *t*-test). NBP14 did not significantly affect T14 expression when comparing conditions and BF areas [Figure 3D; *F*_(5, 70)_ = 2.074, *p* = 0.0790, two-way ANOVA]. However, the treated hemisections showed a continuous T14 decline (Figure 3D), in contrast with the T30 induced profile (Figure 3B). After the co-administration NBP14+T30, we detected a significant interaction between treatment and BF region [Figure 3F; *F*_(5, 35)_ = 3.370, *p* = 0.0137, two-way ANOVA]. In addition, *post-hoc* analysis showed a main effect in the rostral portion, whilst the other slices were not affected when comparing the two conditions (Figure 3F; slice 1, *p* = 0.0109, *t* = 3.435; slice 2, *p* = 0.0779, *t* = 2.064; slice 3, *p* = 0.1538, *t* = 1.599, two tailed paired t-test). T15 did not change the endogenous T14 levels when analyzing the interaction between treatment and anatomical planes [Figure 3H; *F*_(5, 35)_ = 0.6190, *p* = 0.6861, two-way ANOVA].

### Effects of AChE-derived peptides on α7-nAChR

**Figure 4:**
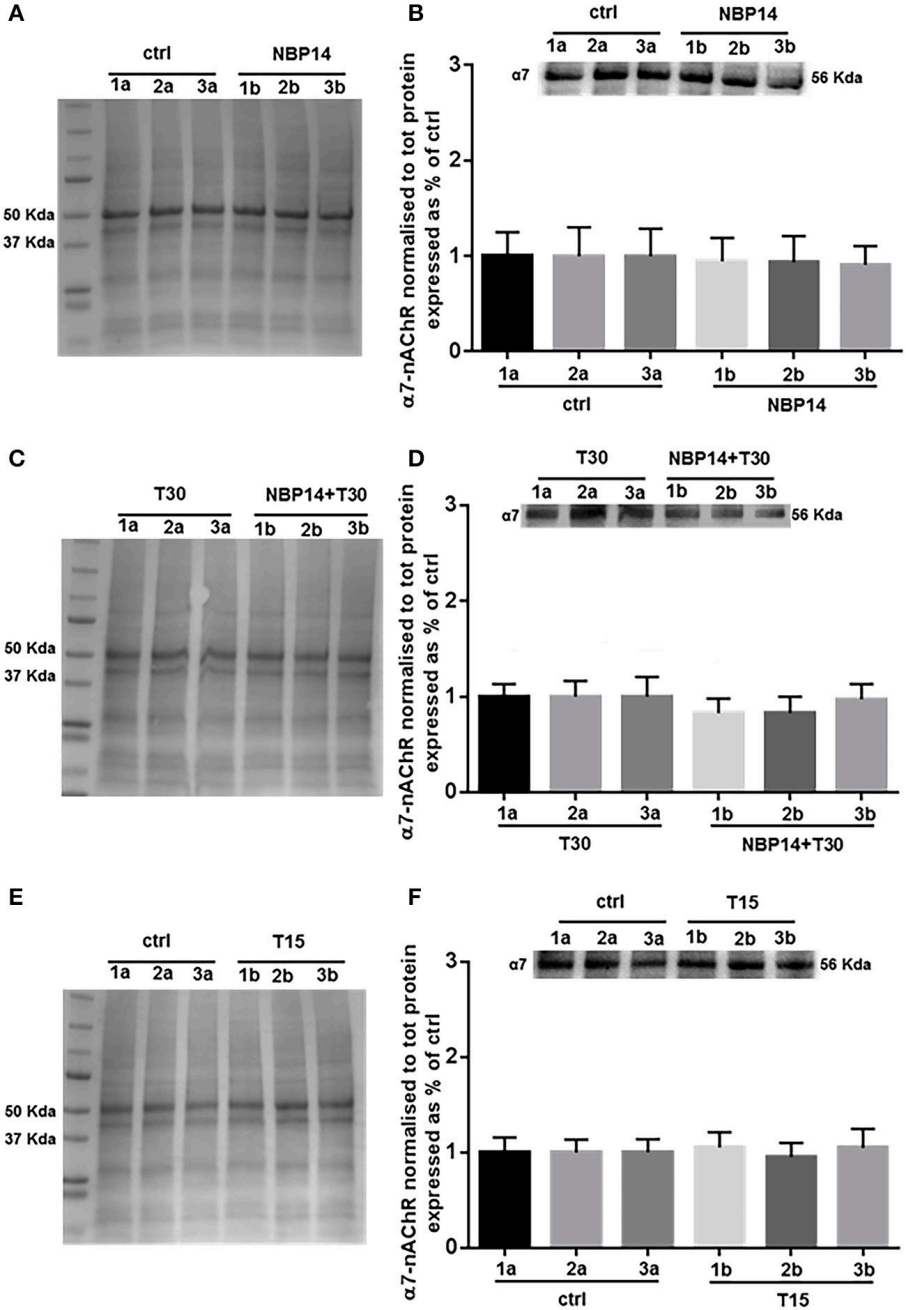
α7-nAChR levels show less variability along the basal forebrain axis following different treatments. **(A,C,E)** Representative total protein staining used as loading control. **(B,D,F)** Representative immunoblots and bar graphs indicating the α7-nAChR levels along the three BF subdivisions. **(A)** Total protein membrane subsequently immunoblotted for α7-nAChR. **(B)** The receptor did not show any rostro-caudal difference between the two conditions [*F*_(5, 55)_ = 0.3227, *p* = 0.8972, two-way ANOVA]. **(C)** Total protein membrane subsequently immunoblotted for α7-nAChR. **(D)** No interaction was observed between conditions and anatomical subdivisions [*F*_(5, 35)_ = 1.503, *p* = 0.2139, two-way ANOVA]. **(E)** Total protein membrane subsequently immunoblotted for α7-nAChR **(F)** No change was detected along the BF anatomical plane after T15 administration [*F*_(5, 35)_ = 0.2600, *p* = 0.9318, two-way ANOVA]. Protein changes were indicated as mean ± SEM. *n* = (hemisections per condition, rats) in **(A,B)** are (36, 12); in **(C,D)** (24, 8); in **(E,F)** (24, 8).

Western blot analysis was performed to determine whether changes in the receptor levels could occur in relation to different treatments and in a site-dependent manner along the BF anatomical planes. NBP14 did not affect the nicotinic receptor expression when evaluating the interaction between conditions and sections [Figure 4B; *F*_(5, 55)_ = 0.3227, *p* = 0.8972, two-way ANOVA]. In line with this observation, also the treatment T30 vs. NBP14+T30 did not induce any difference in α7-nAChR content comparing the investigated BF regions [Figure 4D; *F*_(5, 35)_ = 1.503, *p* = 0.2139, two-way ANOVA]. Following T15 application the receptor was unchanged between conditions and BF subdivisions [Figure 4F; *F*_(5, 35)_ = 0.2600, *p* = 0.9318, two-way ANOVA].

### Effects of AChE-derived peptides on amyloid peptide

**Figure 5:**
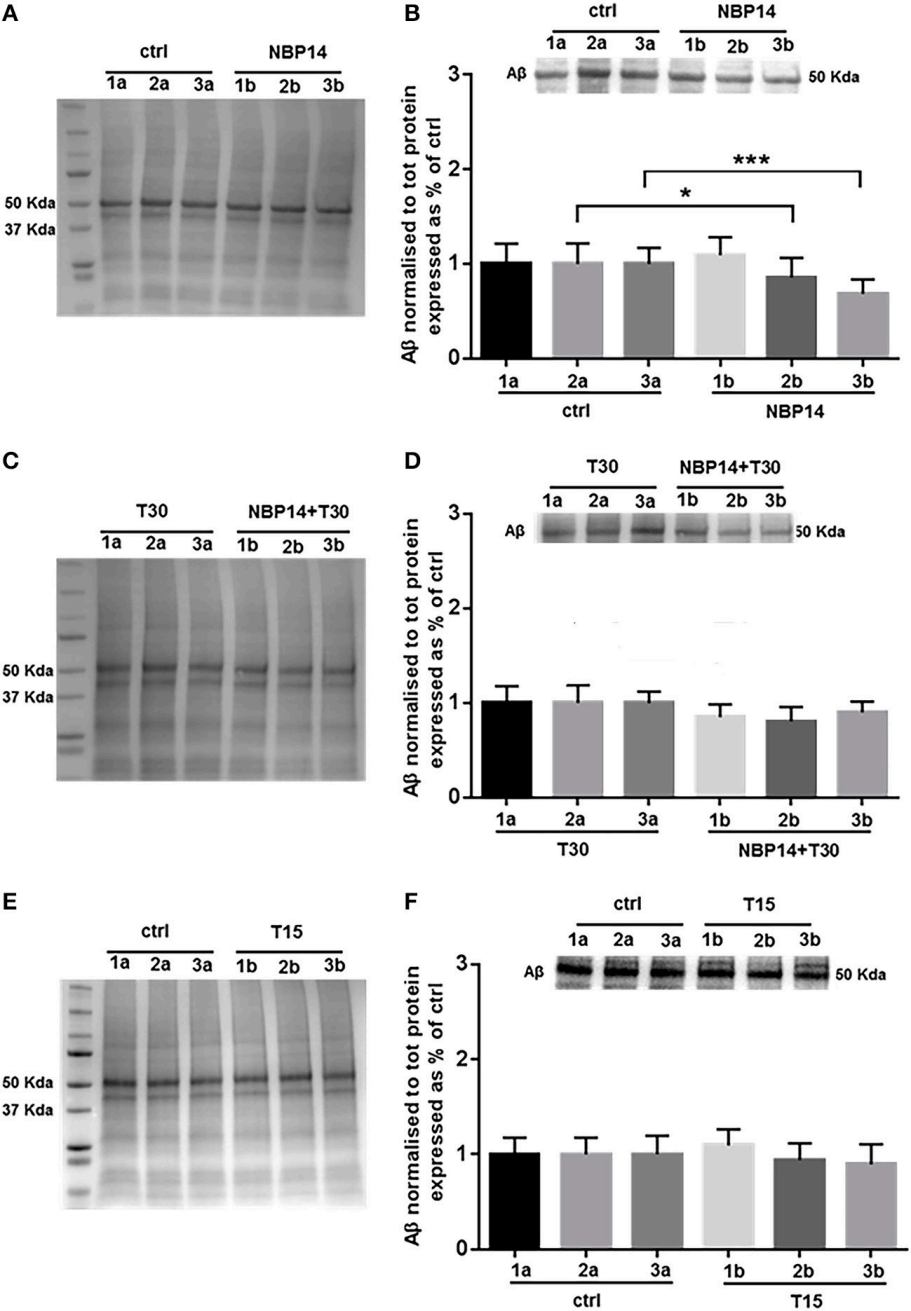
Amyloid beta is specifically modulated by different treatments in the basal forebrain. **(A,C,E)** Representative total protein staining used as loading control. **(B,D,F)** Representative immunoblots and bar graphs indicating Aβ levels along the three BF subdivisions. **(A)** Total protein membrane subsequently immunoblotted for Aβ. **(B)** Amyloid levels were strongly affected between conditions and BF axis [*F*_(5, 55)_ = 7.384, *p* < 0.0001, two-way ANOVA]. A similar expression was detected in the rostral portion (1a and 1b), while the intermediate (2b) and caudal (3b) exposed regions revealed a significant reduction over their untreated sides (2a and 3a) (slice 1, *p* = 0.3756, *t* = 0.9234; slice 2, *p* = 0.0186, *t* = 2.758; slice 3, *p* = 0.0007, *t* = 4.664, two tailed paired *t*-test). **(C)** Total protein membrane subsequently immunoblotted for Aβ. **(D)** The comparison between conditions and anatomical planes did not show a significant interaction [*F*_(5, 35)_ = 1.503, *p* = 0.2139, two-way ANOVA]. **(E)** Total protein membrane subsequently immunoblotted for Aβ. **(F)** T15 triggered no change between conditions and BF planes [*F*_(5, 35)_ = 0.8476, *p* = 0.5255, two-way ANOVA]. Protein changes were indicated as mean ± SEM. *n* = (hemisections per condition, rats) in **(A,B)** are (36, 12); in **(C,D)** (24, 8); in **(E,F)** (24, 8). ^*^p < 0.05, ^***^p < 0.001.

Western blot analysis was carried on to identify whether changes in Aβ levels could occur following different treatments and in a site-specific manner along the BF sections. After administration of the cyclic variant, we observed a substantial interaction between treatment and region [Figure 5B; *F*_(5, 55)_ = 7.384, *p* < 0.0001, two-way ANOVA]. Moreover, *post-hoc* analysis showed that this peptide was gradually declined in the exposed hemisections, being unchanged in the rostral region but significantly reduced in the intermediate and caudal portions over their controls [Figure 5B; slice 1, *p* = 0.3756, *t* = 0.9234; slice 2, *p* = 0.0186, *t* = 2.758; slice 3, *p* = 0.0007, *t* = 4.664, two tailed paired *t*-test]. The combination NBP14+T30 did not affect Aβ expression across the BF axis when compared to T30 [Figure 5D; *F*_(5, 35)_ = 1.503, *p* = 0.2139, two-way ANOVA]. T15 administration did not affect the profile of the amyloid peptide when analyzing conditions and anatomical planes [Figure 5F; *F*_(5, 35)_ = 0.8476, *p* = 0.5255, two-way ANOVA].

### Effects of AChE-derived peptides on phosphorylated Tau

**Figure 6:**
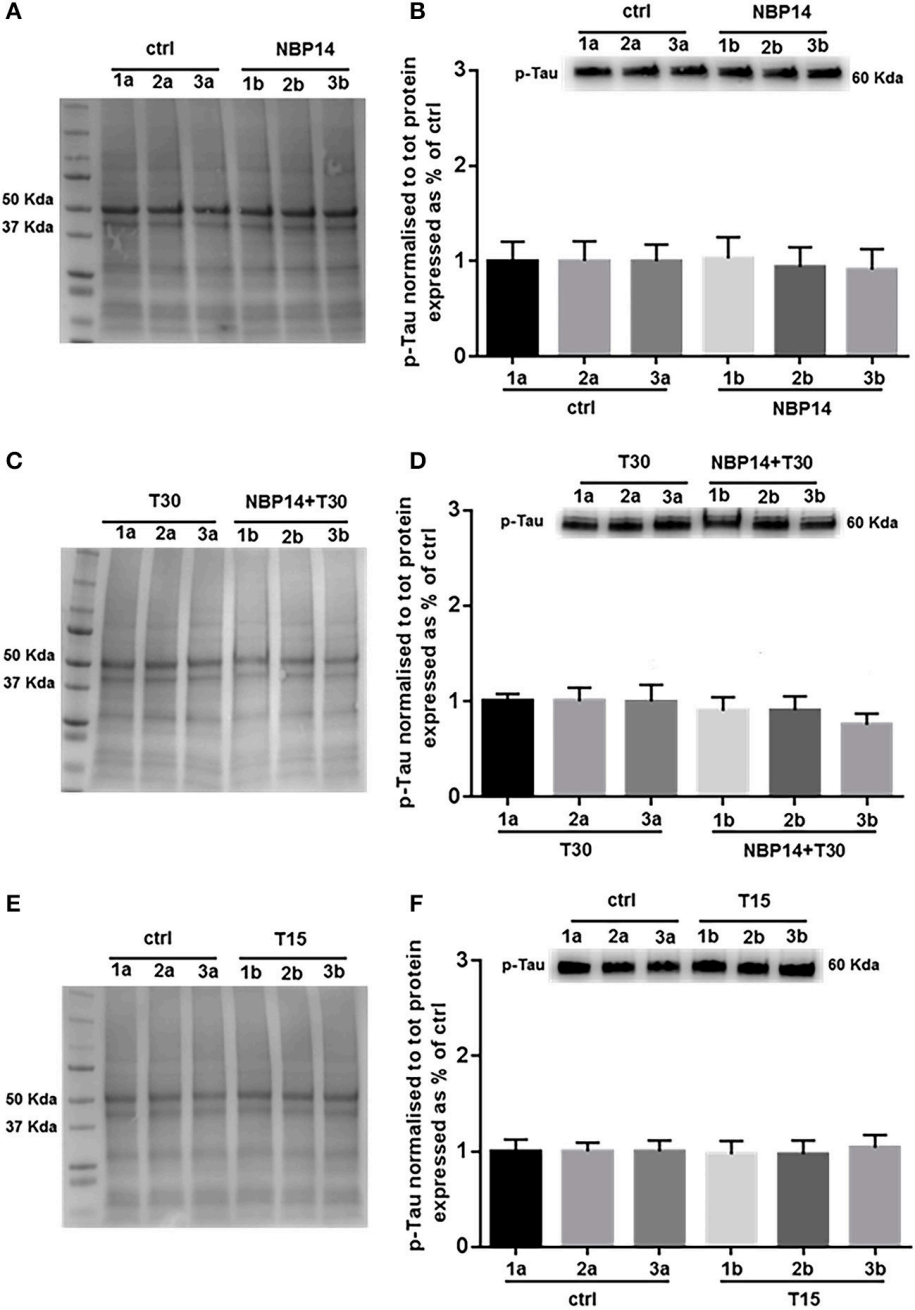
Phosphorylated Tau levels show less variability along the basal forebrain axis following different treatments. **(A,C,E)** Representative total protein staining used as loading control. **(B,D,F)** Representative immunoblots and bar graphs indicating p-Tau levels along the three BF subdivisions. **(A)** Total protein membrane subsequently immunoblotted for p-Tau. **(B)** The BF regions showed no protein difference between conditions [*F*_(5, 55)_ = 0.4774, *p* = 0.7915, two-way ANOVA]. **(C)** Total protein membrane subsequently immunoblotted for p-Tau. **(D)** Phosphorylated Tau was unchanged between conditions and BF axis [*F*_(5, 35)_ = 1.837, *p* = 0.1310, two-way ANOVA]. **(E)** Total protein membrane subsequently immunoblotted for p-Tau. **(F)** Independently from the anatomical area and condition p-Tau showed similar levels [*F*_(5, 35)_ = 0.3212, *p* = 0.8968, two-way ANOVA]. Protein changes were indicated as mean ± SEM. *n* = (hemisections per condition, rats) in **(A,B)** are (36, 12); in **(C,D)** (24, 8); in **(E,F)** (24, 8).

Western blot analysis was carried on to determine whether changes in p-Tau levels could occur following different treatments and in a site-specific manner along the BF axis. NBP14 administration did not triggered any change in p-Tau levels [Figure 6B; *F*_(5, 55)_ = 0.4774, *p* = 0.7915, two-way ANOVA]. Similarly, the simultaneous incubation of the cyclic peptide with T30 did not prompt any substantial difference against the T30 treated group across the BF planes [Figure 6D; *F*_(5, 35)_ = 1.837, *p* = 0.1310, two-way ANOVA]. Consistently with the previous results, T15 did not induce any main effect in p-Tau levels when comparing conditions and regions [Figure 6F; *F*_(5, 35)_ = 0.3212, *p* = 0.8968, two-way ANOVA].

## Discussion

The linear and cyclic AChE-peptides show antagonistic site-selective actions in regulating availability of key neurochemicals linked to Alzheimer's disease.

We have previously described how the linear AChE-peptide may contribute to events underlying neurodegeneration, since (1) is elevated in AD brains (Garcia-Ratés et al., 2016), (2) alters the expression pattern of α7-nAChR, APP, Aβ, p-Tau, and GSK3 (Bond et al., 2009; Garcia-Ratés et al., 2016; Brai et al., 2017), (3) affects calcium influx (Bon and Greenfield, 2003; Greenfield et al., 2004), cell viability (Day and Greenfield, 2003) and AChE release (Day and Greenfield, 2003; Garcia-Ratés et al., 2016), and (4) modulates neuronal activity (Badin et al., 2013, 2016). Conversely, the administration of the cyclic variant, NBP14, reverses the T30 mediated effects both in cell culture and optical imaging experiments (Badin et al., 2016; Garcia-Ratés et al., 2016).

In this study we demonstrated through co-immunoprecipitation assay that T14 and its target receptor form a molecular complex and have continued to characterize NBP14 properties in *ex-vivo* brain slices, since they preserve a 3D anatomical integrity and network activity throughout an extended period (hours). A key aspect in this methodology is to ensure sustained tissue viability, which we have previously confirmed through optical imaging and long-term field potential recordings (Badin et al., 2013, 2016), in line with several decades of electrophysiological reports using this approach (Llinás et al., 1984; Llinás and Greenfield, 1987). The principal goal of this work was to address whether NBP14 could induce an opposite pattern compared to T30 in modulating the levels of the endogenous T14, α7-nAChR, Aβ, and p-Tau, either on its own or in the presence of otherwise toxic exogenous T30.

We explored this possibility testing NBP14 over a longer time period than ever before (Badin et al., 2016) by determining biochemical changes through western blot analysis. Interestingly, we observed that the administration of NBP14, alone or co-applied with T30, evoked a different protein profile along the BF rostro-caudal plane compared to T30, as previously described (Brai et al., 2017). Specifically, T30 promoted a marked site-dependent variation of the nicotinic receptor, amyloid beta, and p-Tau across the basal forebrain subdivisions (Brai et al., 2017). In line with this data, we showed in this study that T30 increased the levels of the endogenous T14. In contrast, NBP14 led to a different protein profile, triggering either a decreased or a similar expression of the investigated neurochemicals across the BF planes. Following NBP14 administration, the most conspicuous changes in protein levels were detected in T14 and amyloid beta. This sensitivity of response could well be related to the abundant extracellular distribution of these proteins after the cleavage from their precursor proteins. On the other hand, the nicotinic receptor and phosphorylated Tau are less affected probably because the modulation of their profile requires a longer exposure due to their transmembrane and intracellular expression respectively.

The opposite effects evoked by the linear and cyclized peptides could be related to (1) their structural conformation, since the peptide cyclization displays elevated stability (Goodwin et al., 2012) compared to the linear counterpart (Howell et al., 2014) and an antagonistic function (Lamberto et al., 2014); (2) their binding affinity with the α7-nAChR, which directly modulates Ca^2+^ permeability (Greenfield and Vaux, 2002; Greenfield et al., 2004; Bond et al., 2009; Garcia-Ratés et al., 2016) and subsequently can affect Aβ and p-Tau pathways; (3) the volume of the BF tissue present in each section; (4) the heterogeneity of cell types, transmitters, neuronal morphology, and distribution of the nicotinic receptor and GSK-3β within the BF (Dinopoulos et al., 1988; Gritti et al., 2006; Mufson et al., 2006). Notably, GSK-3β, which contributes to Aβ production and Tau phosphorylation, is indirectly activated through T30 administration (Bond et al., 2009). In addition, our observations are in line with other studies describing a functional interaction between α7-nAChR, p-Tau, and Aβ and their implication in AD pathobiology (Wang et al., 2000a,b; Nagele et al., 2002; Rubio et al., 2006; Oz et al., 2013; Dineley et al., 2015).

The sequence homology between the AChE-peptide and amyloid beta and their interaction with the nicotinic receptor suggests that these molecules might synergize in modulating cellular cascades either in physiological or pathological conditions. Interestingly, we have previously shown that the co-exposure of Aβ and T30 has an additive effect increasing the AChE activity and reducing cell viability compared to their independent application (Garcia-Ratés et al., 2013). In contrast, the co-administration of NBP14 with T30 or Aβ increased cell viability (Garcia-Ratés et al., 2016), suggesting a potential neuroprotective role of the cyclic variant compared to the aberrant processes driven by T30 or Aβ.

In line with previous reports (Bond et al., 2009; Badin et al., 2016), T15 had no effect on any of the investigated proteins, further validating the bioactive specificity of T30/T14 with the nicotinic receptor.

Taken all together, these data suggest that the AChE-peptides can regulate the processing of the endogenous T14, amyloid beta and phosphorylated Tau, through the link with the alpha7 nicotinic receptor.

## Conclusion

In conclusion, these findings demonstrate that T14 and its target receptor physically interact and provide evidence, from a more physiological preparation and time-frame than previously reported, that NBP14 can displace the natural occurring AChE-peptide from the interaction site of the nicotinic receptor and then prevent potential toxic events through enhanced calcium influx.

These observations further validate that T14 signaling pathway and its interception by NBP14, via competitive displacement, might provide novel key mediators promoting or preventing a neurodegenerative like profile and possibly open up a novel therapeutic strategy.

## Author contributions

EB contributed to design the study, planned and performed the experiments, analyzed the data and wrote the paper. FS and AC performed the experiments. SG designed the study and reviewed the article. All authors read and agreed the final version of the manuscript.

### Conflict of interest statement

SG is the founder and CEO of Neuro-Bio Ltd and holds shares in the company. EB and AC are an employee of the company. FS is a graduate student at the University of Nîmes, France, on industrial placement with the company for 6 months.

## Supplementary material

Supplementary Figure 1 Uncropped original blots showing T14 and α7-nAChR expression and interaction after co-immunoprecipitation in Figure 2. **(A)** Co-IP on *ex-vivo* brain slices and **(B)** whole brain lysate.

Supplementary Figure 2 Uncropped original blots showing T14 expression in Figure 3. **(A)** Ctrl vs. T30, **(B)** Ctrl vs. NBP14, **(C)** T30 vs. NBP14+T30, **(D)** Ctrl vs. T15.

Supplementary Figure 3 Uncropped original blots showing α7-nAChR expression in Figure 4. **(A)** Ctrl vs. NBP14, **(B)** T30 vs. NBP14+T30, **(C)** Ctrl vs. T15.

Supplementary Figure 4 Uncropped original blots showing Aβ expression in Figure 5. **(A)** Ctrl vs. NBP14, **(B)** T30 vs. NBP14+T30, **(C)** Ctrl vs. T15.

Supplementary Figure 5 Uncropped original blots showing p-Tau expression in Figure 6. **(A)** Ctrl vs. NBP14, **(B)** T30 vs. NBP14+T30, **(C)** Ctrl vs. T15.
