# Supplementary figures and images for: Modulatory Effects of a Novel Cyclized Peptide in Reducing the Expression of Markers Linked to Alzheimer's Disease
Source: Front Neurosci. 2018 Jun 13;12:362. doi: 10.3389/fnins.2018.00362 (PMC6008575; doi:10.3389/fnins.2018.00362)

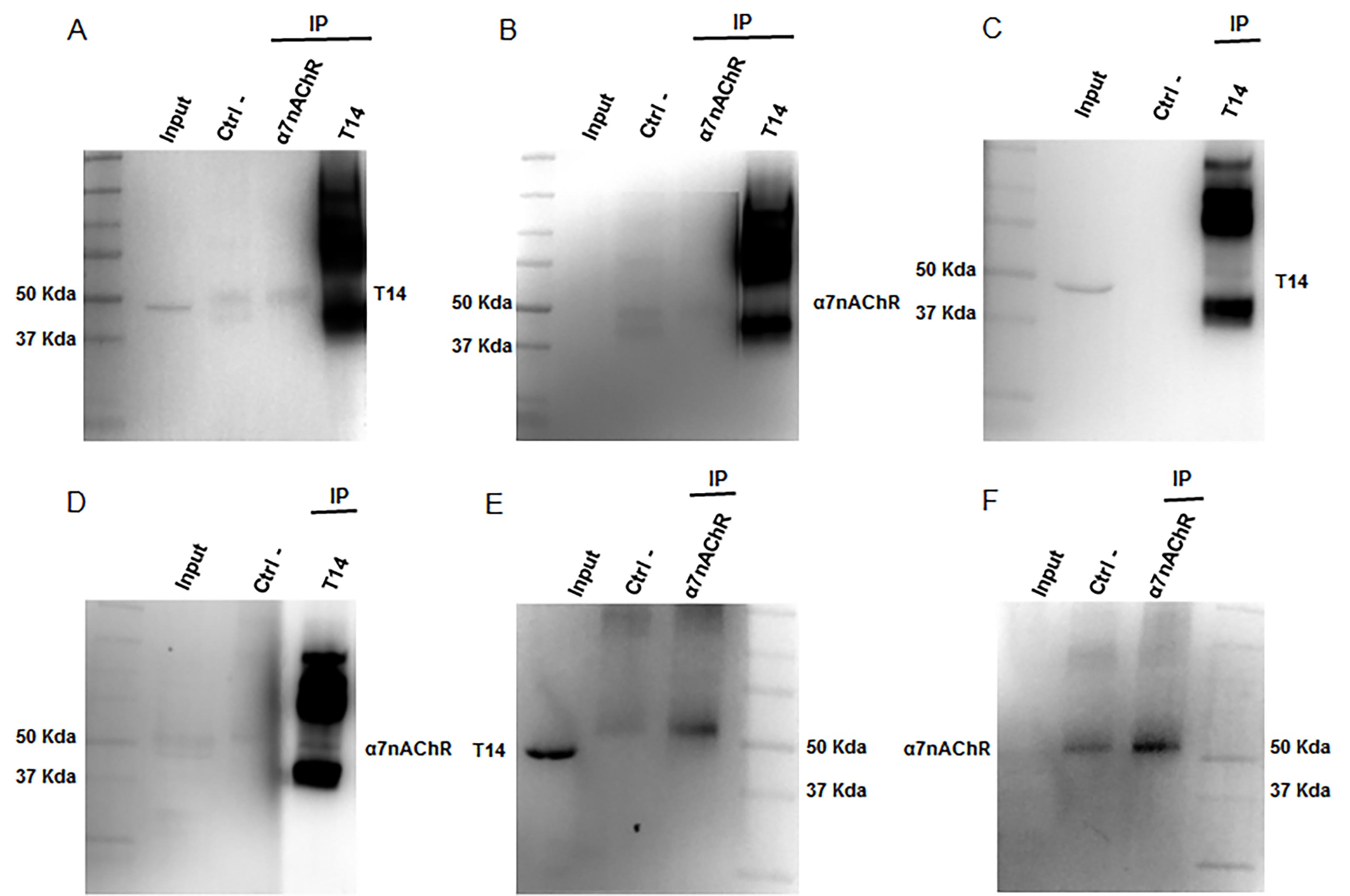

Supplement: Supplementary Figure 1 — Uncropped original blots showing T14 and α7-nAChR expression and interaction after co-immunoprecipitation in Figure 2. (A) Co-IP on ex-vivo brain slices and (B) whole brain lysate. [file Image_1.JPEG]

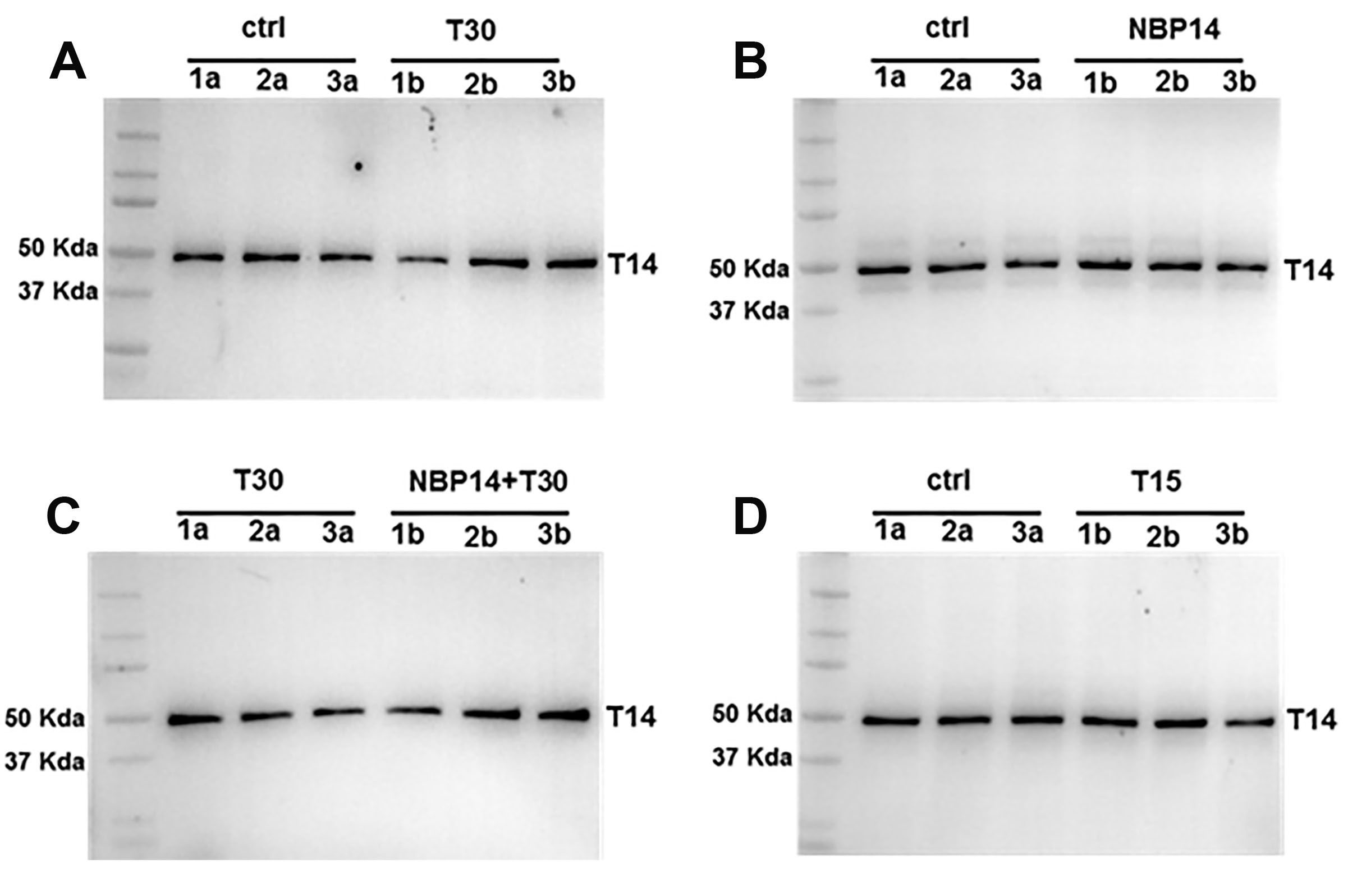

Supplement: Supplementary Figure 2 — Uncropped original blots showing T14 expression in Figure 3. (A) Ctrl vs. T30, (B) Ctrl vs. NBP14, (C) T30 vs. NBP14+T30, (D) Ctrl vs. T15. [file Image_2.JPEG]

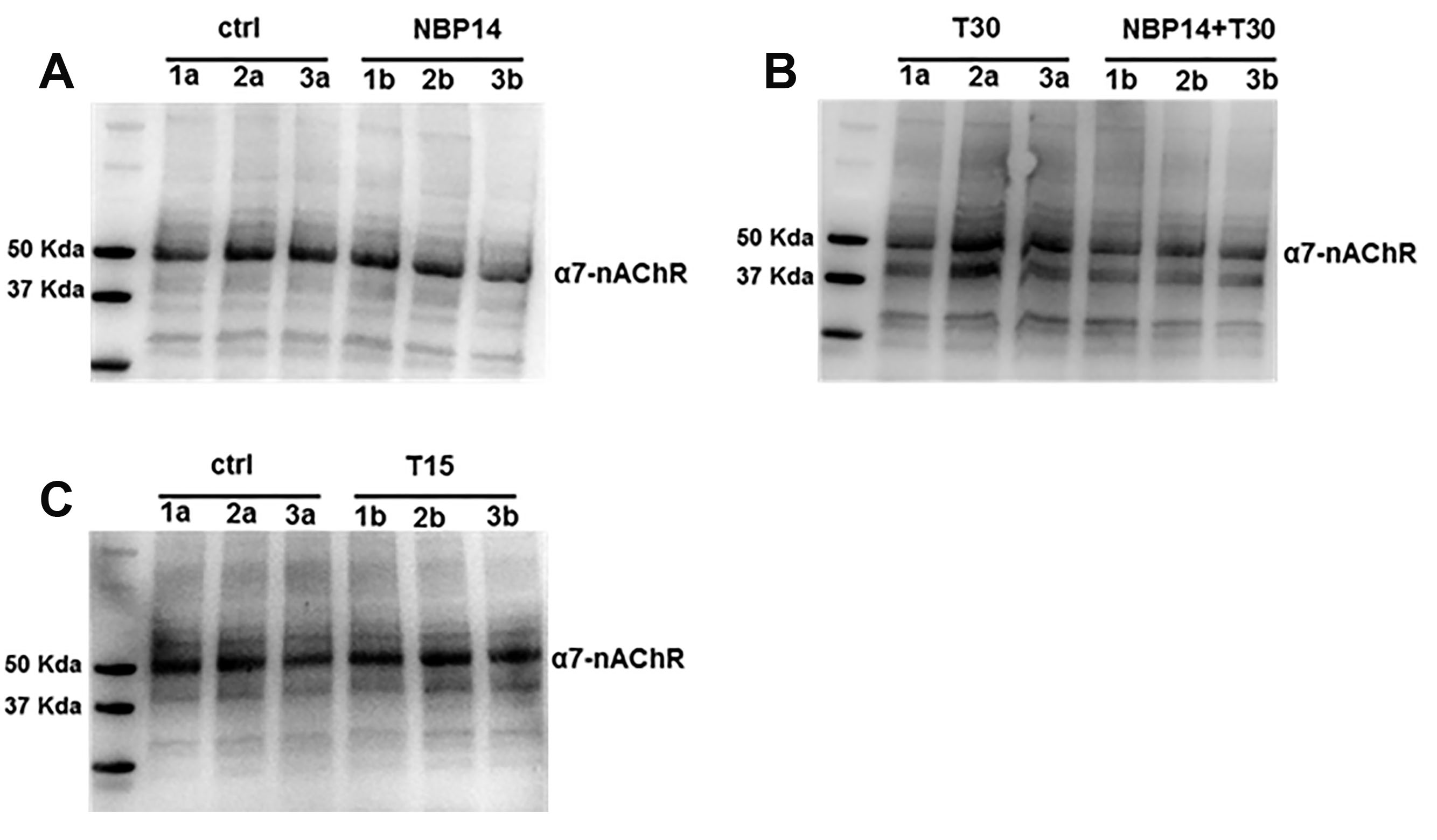

Supplement: Supplementary Figure 3 — Uncropped original blots showing α7-nAChR expression in Figure 4. (A) Ctrl vs. NBP14, (B) T30 vs. NBP14+T30, (C) Ctrl vs. T15. [file Image_3.JPEG]

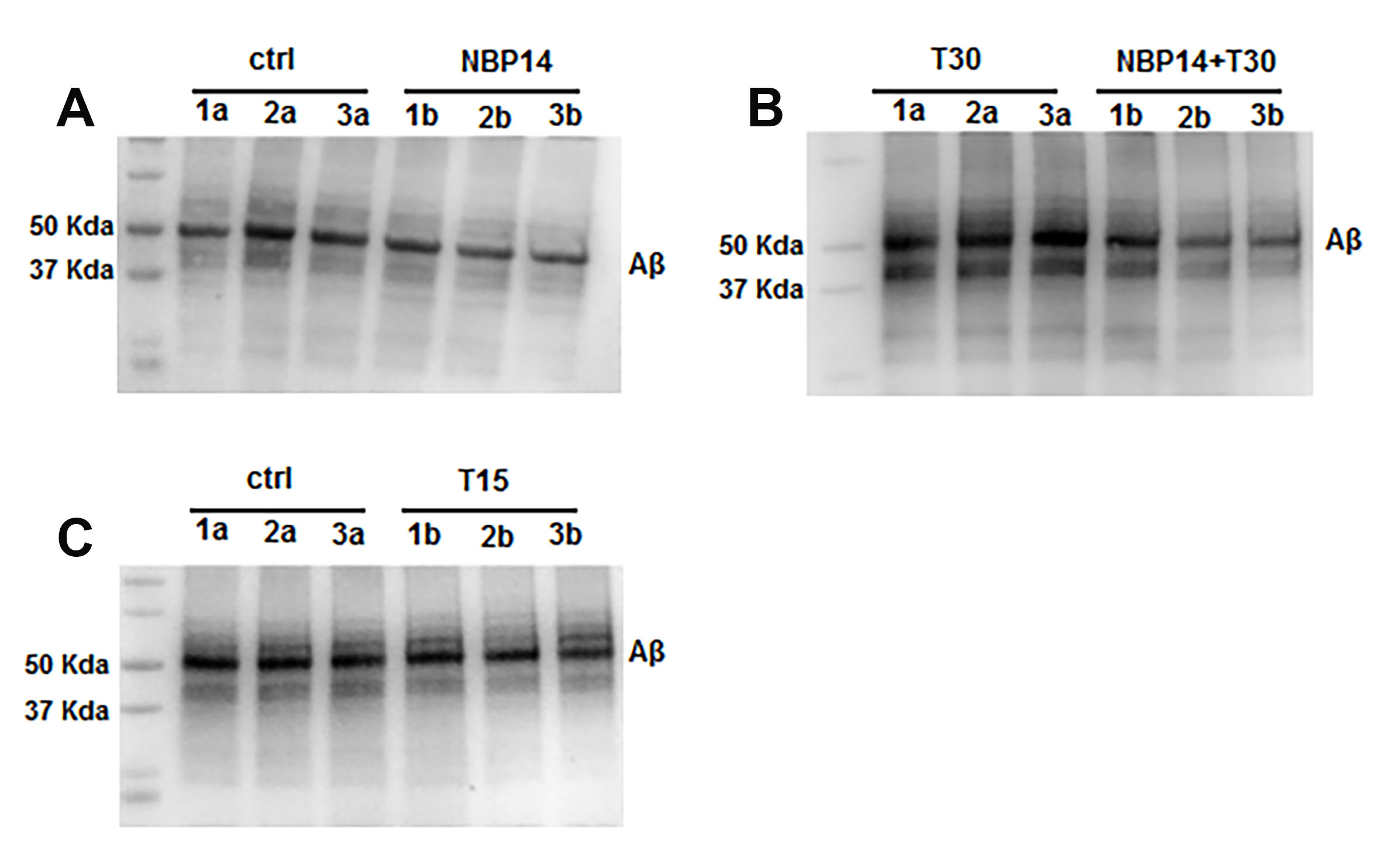

Supplement: Supplementary Figure 4 — Uncropped original blots showing Aβ expression in Figure 5. (A) Ctrl vs. NBP14, (B) T30 vs. NBP14+T30, (C) Ctrl vs. T15. [file Image_4.JPEG]

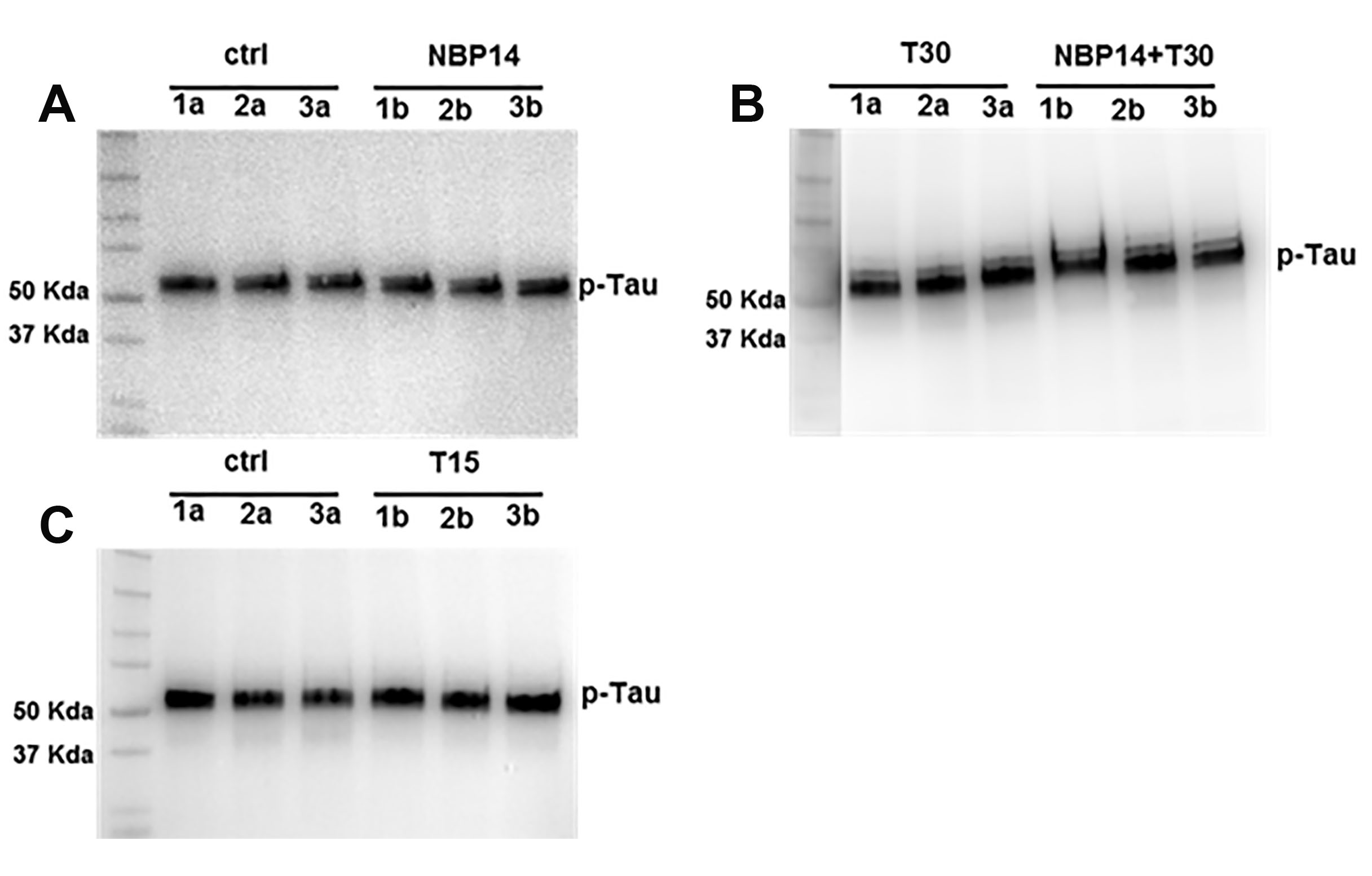

Supplement: Supplementary Figure 5 — Uncropped original blots showing p-Tau expression in Figure 6. (A) Ctrl vs. NBP14, (B) T30 vs. NBP14+T30, (C) Ctrl vs. T15. [file Image_5.JPEG]
